# Self versus Environment Motion in Postural Control

**DOI:** 10.1371/journal.pcbi.1000680

**Published:** 2010-02-19

**Authors:** Kalpana Dokka, Robert V. Kenyon, Emily A. Keshner, Konrad P. Kording

**Affiliations:** 1 Department of Anatomy and Neurobiology, Washington University, Saint Louis, Missouri, United States of America; 2 Department of Computer Science, University of Illinois at Chicago, Chicago, Illinois, United States of America; 3 Department of Physical Therapy, Temple University, Philadelphia, Pennsylvania, United States of America; 4 Department of Electrical and Computer Engineering, Temple University, Philadelphia, Pennsylvania, United States of America; 5 Department of Physical Medicine and Rehabilitation, Rehabilitation Institute of Chicago, Chicago, Illinois, United States of America; University College London, United Kingdom

## Abstract

To stabilize our position in space we use visual information as well as non-visual physical motion cues. However, visual cues can be ambiguous: visually perceived motion may be caused by self-movement, movement of the environment, or both. The nervous system must combine the ambiguous visual cues with noisy physical motion cues to resolve this ambiguity and control our body posture. Here we have developed a Bayesian model that formalizes how the nervous system could solve this problem. In this model, the nervous system combines the sensory cues to estimate the movement of the body. We analytically demonstrate that, as long as visual stimulation is fast in comparison to the uncertainty in our perception of body movement, the optimal strategy is to weight visually perceived movement velocities proportional to a power law. We find that this model accounts for the nonlinear influence of experimentally induced visual motion on human postural behavior both in our data and in previously published results.

## Author Summary

Visual cues typically provide ambiguous information about the orientation of our body in space. When we perceive relative motion between ourselves and the environment, it could have been caused by our movement within the environment, or the movement of the environment around us, or the simultaneous movements of both our body and the environment. The nervous system must resolve this ambiguity for efficient control of our body posture during stance. Here, we show that the nervous system could solve this problem by optimally combining visual signals with physical motion cues. Sensory ambiguity is a central problem during cue combination. Our results thus have implications on how the nervous system could resolve sensory ambiguity in other cue combination tasks.

## Introduction

Our visual system senses the movement of objects relative to ourselves. Barring contextual information, a car approaching us rapidly while we stand still may produce the same visual motion cues as if we and the car were approaching each other. The nervous system thus needs to deal with this problem of ambiguity which will be reflected in the way we control our body posture [1]–[3]. Consequently, neuroscientists have extensively studied such situations. In such studies, a subject typically stands in front of a visual display and postural reactions to varied movements of the displayed visual scene are measured [4]–[11]. Even in the absence of direct physical perturbations, subjects actively produce compensatory body movements in response to the movement of the visual scene. This indicates that subjects attribute part of the visual motion to their own body while they resolve the ambiguity in visual stimuli.

**Figure 1:**
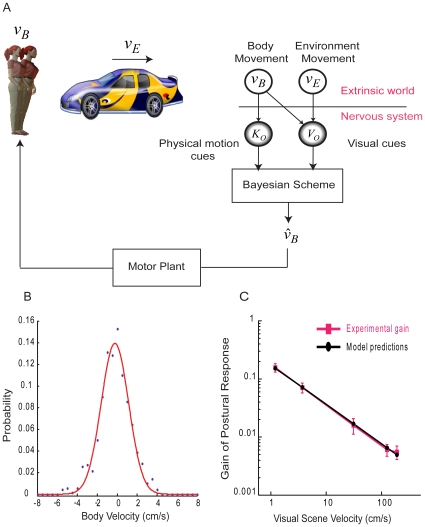
Sensory ambiguity influences postural behavior. (A) Graphical model, a compact way of describing the assumptions made by a Bayesian model. 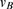 is the velocity of body motion, while 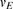 is the velocity of the environment motion. 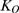 represents a noisy estimate of the body velocity that is sensed by kinesthetic and vestibular signals. 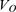 represents the visually perceived velocity of the relative motion between the body and the environment. The attribution model estimates 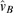 from these perceived cues. (B) Distribution of body velocities during unperturbed stance averaged across subjects tested in our experiment (C) Experimental data and model fits for healthy subjects tested in our experiment.

Here we constructed a Bayesian attribution model (Fig. 1A) to examine how the nervous system may solve this problem of sensory ambiguity. This model shows that optimal solutions will generally take on the form of power laws. We found that the results from experiments with both healthy subjects and patients suffering from vestibular deficits are well fit by power laws. The nervous system thus appears to combine visual and physical motion cues to estimate our body movement for the control of posture in a fashion that is close to optimal.

## Results

**Figure 2:**
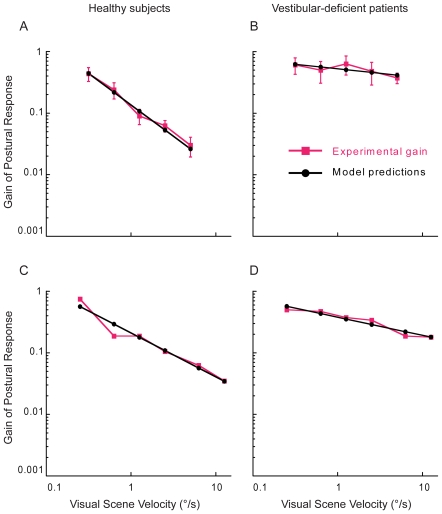
Gain of the postural response of healthy subjects and patients with vestibular loss. (A) and (B) represent the experimental data and model fits of healthy subjects and patients tested in ref. 5 (Mergner et al. 2005). (C) and (D) represent the experimental data and model fits of healthy subjects and patients tested in ref. 4 (Peterka and Benolken 1995).

To test our Bayesian attribution model, we considered data from two published experiments with healthy subjects [4],[5] as well as a new experiment we performed to cover the range of visual scene velocities that are relevant to the model predictions. Any purely linear model, for example a Kalman controller, predicts that the gain of the postural response, which is the influence of visual scene motion on the amplitude of postural reactions, remains constant. For these datasets, however, the gain of the postural response decreased with increasing velocities of visual scene motion (Fig. 1C, 2A and 2C; slope = −0.78±0.15 s.d. across datasets, *p*<0.005). At low velocities, the gain was close to one which would be expected if the nervous system viewed the body as the sole source of the visually perceived motion. At higher velocities though, the gain decreased which would be expected if the nervous system no longer attributed all of the visually perceived motion to the body. The nervous system thus does not appear to simply assume that visually perceived motion can be fully attributable to the body.

To explain this nonlinear influence of visual scene velocity on the postural response, we constructed a model that describes how the nervous system could solve the problem of sensory ambiguity (Fig. 1A). The nervous system can combine visual cues with physical motion cues, such as vestibular and kinesthetic inputs, to estimate our body movement [12]–[16]. However, our sensory information is not perfect and recent studies have emphasized the importance of uncertainty in such cue combination problems [17]–[19]. Visual information has little noise when compared with physical motion cues [20]. However, it is ambiguous as it does not directly reveal if the body, the environment or both are the source of the visually perceived movement. In comparison to visual cues, physical motion cues are typically more noisy but they are not characterized by the same kind of ambiguity. For these reasons, the nervous system can never be certain about the velocity of the body movement, but can at best estimate it using principles of optimal Bayesian calculations [21]–[25]. To solve the ambiguity problem, the model estimated the velocity of body's movement for which the perceived visual and physical motion cues were most likely.

Such estimation is only possible if the nervous system has additional information about two factors: typical movements in the environment and typical uncertainty about body movements [26]. For example, if a car sometimes moves fast and our body typically moves slowly, then the nervous system would naturally attribute fast movement to the car and slow movement to our body. Indeed, recent research has indicated that human subjects use the fact that slow rather than fast movements are more frequent in the environment when they estimate velocities of moving visual objects [27]–[30]. This distribution, used by human subjects, is called a prior. Following these studies our model used a sparse prior for movements in the visual environment, that is a prior which assigns high probability to slower movements in the environment and low probability to faster movements in the environment [29].

We wanted to estimate the form of the prior over body movements from our experimental data. We found that when subjects maintained an upright body posture while viewing a stationary visual scene, the distribution of their body velocity was best described by a Gaussian (Fig. 1B). Therefore, we used a Gaussian to represent the prior over body velocity.

The attribution model derives from five assumptions. We assume the above sparse prior over movements in the environment [29]. We assume that for the movement of visual environment that is vivid and has high contrast, visual cues provide an estimate of relative movement that has vanishing uncertainty. We assume a Gaussian for the prior over body movement (see Methods for details). We also assume a Gaussian for the likelihood of the physical motion cues which indicate that the body is not actually moving and is close to the upright position. Lastly we assume that visual scene velocities are large in comparison to the uncertainty in our detection of our body movements [31]. Under these assumptions, we can analytically derive that the best solution has a gain that varies as a power law with the visual scene velocity (see Methods for details). We thus obtain a compact, two parameter model that predicts the influence of visual perturbations on the estimates of body movement.

Our attribution model calculates how the nervous system should combine information from visual and physical senses to optimally estimate the velocity of body movement. However, the nervous system does not need to solve its problems in an optimal way, but may use simple heuristics [32]. We thus proceeded to compare the attribution model with other models in its ability to explain the decrease in the gain of postural reactions. For this purpose, we compared models using the Bayesian Information Criterion (BIC) which is a technique that allows the comparison of models with different numbers of free parameters [33]. For the gains observed in our experiment (Fig. 1C), the Bayesian model had a BIC of −7.5±1.84 (mean BIC±s.e.m. across subjects). We found that a linear model that predicted constant gain of postural reactions could not explain the observed results (BIC = 1.08±0.59, *p*<0.001, paired *t*-test between BIC values).

We then considered a model in which the amplitude of postural response increased logarithmically up to a threshold stimulus velocity and then saturated. This model predicted the response gains observed at higher scene velocities more poorly than the attribution model (BIC = −3.45±0.99, *p*<0.05). We also tested another model in which the gain was initially constant but decreased monotonically with increasing visual scene velocities. This model did worse at predicting the gain than the Bayesian model (BIC = 5.82±0.04, *p*<0.001). Thus, the Bayesian model that estimated the velocity of the body movement best fit the available data.

Another way of applying the attribution model is to human behavior in disease states. Patients with bilateral vestibular loss have vestibular cues of inferior quality [34]. The attribution model suggests that these patients' postural behavior would be based more strongly on visual feedback and that their gain should decrease less steeply as a function of stimulus velocity. Indeed, patients tested in previous studies [4],[5] showed a greater influence of vision on posture and gains that decreased less steeply (Fig. 2B, 2D slope = −0.22±0.1 s.d. across datasets, *p*<0.005) when compared with healthy subjects, a phenomenon that is well mimicked by the attribution model.

The postural behavior of patients showed marked differences from that of healthy subjects [4]. At low visual scene velocities, patients and healthy subjects had similar gain values. However, at higher scene velocities, patients exhibited larger gains when compared with healthy subjects. If the postural responses in patients were only influenced by elevated noise in the vestibular channels, the gain should vary in a similar manner at all visual scene velocities. That is, the gain of patients should be higher than healthy subjects at all visual scene velocities. However, increased gain of patients only at higher scene velocities alludes to a change in how patients interact with large movements in the visual environment. In our model, the best fit to the data of healthy subjects corresponds to a prior of about 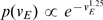, while the fit to the patients' data corresponds to a prior of 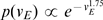 (see Methods for details). It would thus appear that rather than a sparse prior, patients have a prior that is closer to a Gaussian. It is not surprising that patients interact with the extrinsic environment differently from healthy subjects. In fact, such patients can develop space and motion phobia particularly in situations where there is a conflict between visual and vestibular cues and may actively avoid such conflicting environments [35]–[37]. Our model fits suggest that patients may seek out environments that are devoid of fast movement of large field stimuli. This is a prediction that can be tested in future research, for example by equipping patients with telemetric devices with cameras that record velocities in their environment.

## Discussion

When we visually perceive displacement between ourselves and the environment, it may be caused by the movement of our body, movement of the environment, or both. In this paper, we have presented a model that formalizes how the nervous system could solve the problems of both ambiguity (self vs environment) and noise in perceived sensory cues. We suggest that the nervous system could solve these problems by estimating the movement of the body as per the principles of Bayesian calculations. We found that the model can account for the gain of postural responses when both healthy subjects and patients with vestibular loss viewed movement of a visual scene at various velocities. Importantly, our model predicts a simple functional form, power laws, as the best cue combination strategy. This makes it easy to test predictions without having to implement complicated estimation procedures.

Postural stabilization during stance is a two-step process comprised of estimation and control and in this paper we have only focused on estimation. Computational models in the past have examined how the nervous system implements this two-step process and have explained a wide range of data [5], [8], [34], [38]–[40]. In these models, cue combination was implemented as a change in the sensory weights [8],[14] and incorporation of nonlinear elements [5],[34],[39]. The control aspect was typically implemented by approximating the human body to a single- or double-link inverted pendulum, linearized about the upright position. These models are powerful tools for describing human behavior as they can describe changes both in amplitude and in phase as stimulus parameters are varied. As current models already largely separate postural control into an estimation part and an estimation-dependent control part, it would be straightforward to combine our estimation system with a dynamical control system.

When the control strategy is linear then any nonlinearity has to come from the estimation stage. If control is nonlinear, then there will be interactions between nonlinearities in estimation and control. Our attribution model exclusively focuses on the source of the nonlinearity inherent in the estimation process. If control is nonlinear then parts of the effects we describe here may be due to nonlinearities in control and parts due to estimation. The influence of the nonlinearity in each could be tested by experiments that decouple estimation from control. Importantly, though past models have assumed nonlinearities in the estimation part of the model [5],[8],[14],[34], we give a systematic reason for why this nonlinearity should exist and why it should have approximately the form that has been assumed in past studies.

To test our model, we used visual scene velocities that were in all likelihood, larger than the uncertainty in our perception of our body sway. Our model analytically demonstrates that for these velocities, the gain is proportional to a power law over the visual scene velocity. This leads us to question how the model would perform over a different range of scene velocities. There could be two possible solutions to this question. Firstly, the nervous system may use power laws to estimate the gain of the postural responses at all visual scene velocities. However, this solution does not make any sense as it would predict infinite gain near zero velocity. Secondly, at very small scene velocities, the nervous system may adopt a strategy different from power laws. We argue in favor of the latter possibility. We predict that at scene velocities that are close to our perceptual threshold of body sway, our attribution model would fail to explain the gain of postural responses. In this situation, the Taylor series expansion that we use can no longer be truncated after the first term and quadratic elements need to be considered (see Methods). The attribution model will predict power laws if the prior over visual movements is locally smooth within the range of uncertainty in our perception of body movement.

Ambiguity is a central aspect of various cue combination problems in perception and motor control and here we have characterized its influence on postural control. The success of the attribution model in predicting human behavior suggests that the nervous system may employ simple schemes, such as power laws, to implement the best solution to the problem of sensory ambiguity. While recent research indicates how the nervous system could integrate cues that have Gaussian likelihoods [41] or priors [29], little is known about the way non-Gaussian probability distributions may be represented at the neuronal level. The nonlinearity in cue combination that we observed here raises interesting questions about the underlying neural basis of these computations in the nervous system.

## Methods

### Ethics statement

Ten healthy young adults (age: 20–34 years) participated in our experiment. Subjects had no history of neurological or postural disorders and had normal or corrected-to-normal vision. Subjects were informed about the experimental procedures and informed consent was obtained as per the guidelines of the Institutional Review Board of Northwestern University.

### Experimental setup

A computer-generated virtual reality system was used to simulate the movement of the visual environment. Subjects viewed a virtual scene projected via a stereo-capable projector (Electrohome Marquis 8500) onto a 2.6 m×3.2 m back-projection screen. The virtual scene consisted of a 30.5 m wide by 6.1 m high by 30.5 m deep room containing round columns with patterned rugs and painted ceiling. Beyond the virtual scene was a landscape consisting of mountains, meadows, sky and clouds. Subjects were asked to wear liquid crystal stereo shutter glasses (Stereographics, Inc.) which separated the field sequential stereo images into right and left eye images. Reflective markers (Motion Analysis, Inc.) attached to the shutter glasses provided real-time orientation of the head that was used to compute correct perspective and stereo projections for the scene. Consequently, virtual objects retained their true perspective and position in space regardless of the subject's movement.

Subjects stood in front of the visual scene with their feet shoulder-width apart and their arms bent approximately 90° at their elbows. The location of subjects' feet on the support surface was marked; subjects were instructed to stand at the same location at the beginning of each trial. During each trial, subjects were instructed to maintain an upright posture while looking straight ahead at the visual scene. Subjects viewed anterior-posterior sinusoidal oscillation of the scene at 0.2 Hz and 5 peak amplitudes: 1, 3, 25, 100 and 150 cm. The visual scene thus oscillated at peak velocities of 1.2, 3.7, 31, 125 and 188 cm/s, respectively. Subjects viewed each scene velocity once for a period of 60 s in random order. In addition, subjects experienced a control condition in which they viewed the stationary visual scene.

Reflective markers were placed on the shoulder joints and fifth lumbar vertebra. A six infra-red camera (Motion Analysis, Inc.) system was used to record the displacement of the reflective markers at 120 Hz. Displacement data of the markers was low pass filtered using a fourth order Butterworth digital filter with a cutoff at 6 Hz. Trunk displacement, chosen as an indicator of postural response, was calculated using the displacement of the shoulder and spine markers [42]. Amplitude of the postural response at the frequency of the visual scene motion, that is 0.2 Hz, was calculated in a manner adopted in neurophysiological studies [43],[44]. A sinusoid of frequency 0.2 Hz was chosen. The amplitude and the phase of this sinusoid were estimated such that the squared error between the trunk displacement and the fitted sinusoid was minimized. The amplitude of the fitted sinusoid thus indicated the amplitude of the postural response at the frequency of the visual scene motion. The gain of the trunk displacement was then computed as the ratio of the amplitude of the fitted sinusoid to the amplitude of visual scene motion.

### Bayesian model of ambiguity resolution

We formalize the ambiguity problem encountered by the nervous system with the help of a graphical model (Fig. 1A). The visual scene projected on the display sinusoidally oscillates with a velocity 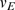, while the velocity of the body movement is 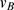. 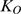 represents a noisy estimate of body velocity that is sensed by vestibular and kinesthetic signals. On the other hand, 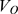 represents the visually perceived velocity of the relative movement between the body and the environment. Our Bayesian model combines the sensory cues, 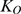 and 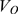, to obtain the best estimate of body velocity, 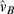. As the amplitude of postural reactions are influenced by subject's perceived body movement [2],[45], we assume that the nervous system produces body movements proportional to the estimated body velocity 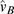.

Using Bayes' rule we obtain:
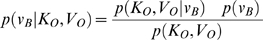
(1)


We assume that the visual and physical channels are affected by independent noise. Therefore, we get:
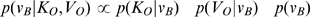
(2)


We estimated the form of the prior over body velocity, 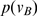, from our data. In our experiment, subjects experienced a control condition where they maintained upright body posture when viewing a stationary visual scene. We computed the average velocity of the trunk displacement across all subjects [42]. We then computed a histogram of the body velocity and observed that a Gaussian best described the distribution of body velocity (Fig. 1B). We, therefore, assumed that subjects prior over body movements would be represented by a Gaussian. While the actual body movements during unperturbed stance are large, the more relevant information is the underlying uncertainty in our perception of our body sway. The uncertainty in our perception of our body sway is much narrower than the width of the distribution of the actual body velocities seen in Fig. 1B [31]. This is because for small body movements during normal stance, the nervous system may not constrain the body even though it is aware that the body has moved away from the upright position [46].

As the likelihood of the physical motion cues, 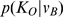, can also be represented by a Gaussian, we define:
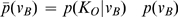
(3)Here 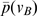 represents a Gaussian for the combined prior-and-likelihood with variance 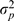.

The likelihood of visual motion cues, 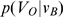, is given by:
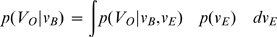
(4)Humans expect visual objects in their environment to move slowly more often than rapidly. This bias has been interpreted as a prior in a Bayesian system. We therefore use a sparse prior of the functional form 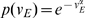 [29]. As visual cues are precise when compared with other sensory cues, we assume that the variance of the noise in visual channels is negligible. Furthermore, in the experimental situations we model here, movement of the visual display is relatively fast in comparison to the typical uncertainty subjects may have about their body velocity [31].

We therefore marginalize over all possible 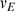 to obtain:
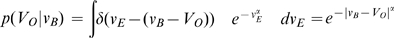
(5)Substituting Equations 3 and 5 in Equation 2, we get:
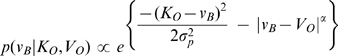
(6)


In the situations we model here, subjects stood on a stationary support surface. Thus, the physical motion cues indicated that the body was close to the upright position; that is 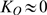.

We therefore get:
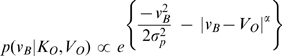
(7)


For body movements close to the upright position, we can use a Taylor series expansion and drop elements of order 2 and higher to solve the second exponent term in Equation 7. We thus get:
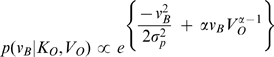
(8)


Importantly, when visual scene velocities are large in comparison to the typical uncertainty in our perception of our body movements, then the maximum of the (visual) environmental prior is far away. As that is far away and the uncertainty in the perception of body movement is narrow, the approximation that only zero- and first-order terms will be important is well justified.

The resulting estimate represents a Gaussian with a maximum at:
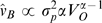
(9)Thus, the best estimate of the body velocity 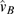, as long as the environment velocity is large in comparison to the typical uncertainty in our perception of body sway, can be represented as a power law over the environment velocity 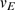.



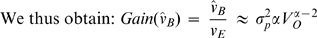
(10)Our model thus has two free parameters: 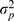 the variance of the noise in prior-and-likelihood of the physical motion cues; 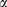, the parameter associated with the prior over environmental velocities.

We fitted the model (Equation 10) to the experimentally measured gain of healthy subjects tested in our experiment. We then fitted the model to the experimentally measured gains of healthy subjects and vestibular-deficient patients tested in previous studies [4],[5]. We chose the model parameters such that the mean squared error between the model fits and the experimental data was minimized.

For healthy subjects, the values of free parameters were as follows: 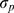 = 0.34 and 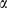 = 1.32 (for subjects tested in our experiment); 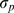 = 0.37 and 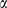 = 1.28 (for subjects tested by Peterka et al.); 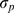 = 0.33 and 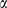 = 1.03 (for subjects tested by Mergner et al.). For vestibular-deficient patients, the values of free parameters were as follows: 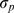 = 0.46 and 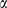 = 1.7 (for patients tested by Peterka et al.); 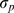 = 0.524 and 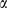 = 1.85 (for patients tested by Mergner et al.).

### Model comparisons

To test the performance of our attribution model, we compared it with other simple models of postural control.

**Figure 3:**
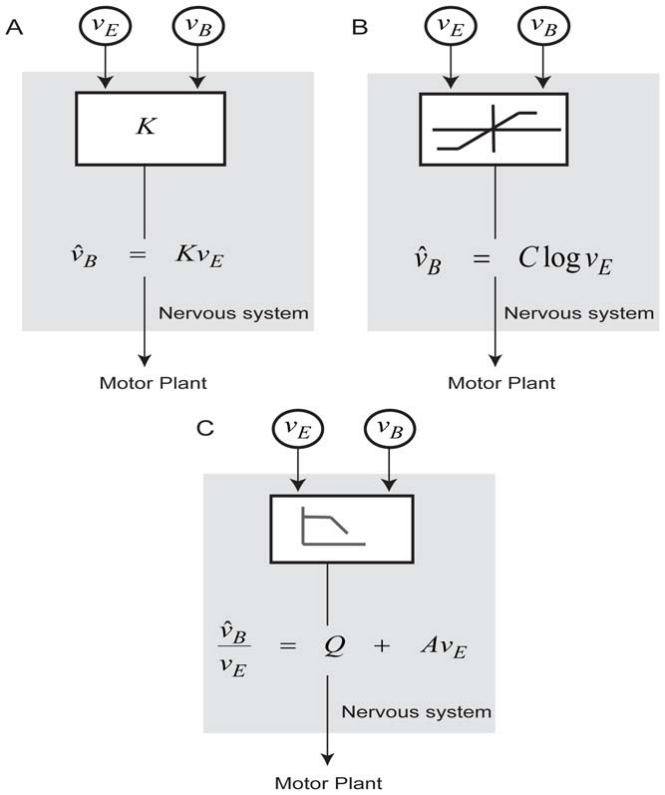
Alternative models of postural control. (A) Model that predicts constant gain of the postural response. (B) Model where amplitude of the postural response increases logarithmically with visual scene velocity and then saturates. (C) Model where the gain is initially constant and then monotonically decreases with the visual scene velocity. Here 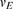 represents the visual scene velocity, 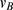 represents the true body velocity and 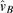 indicates the estimate of the body velocity calculated by the models.

We first considered a linear model in which the gain of postural response was constant (Fig. 3A). This model had a single free parameter, the gain 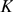, and had a functional form:
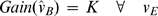



We then developed a nonlinear model that incorporated the findings of published empirical and modeling studies. The amplitude of postural reaction is known to increase logarithmically with the visual scene velocity until it saturates [4]. We tested a model of the functional form (Fig. 3B):
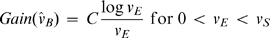


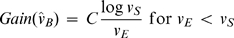
Here 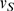 represents the visual scene velocity at which saturation occurs. We chose 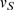 = 2.8 cm/s based on the previous findings in the literature [5]. This model had a single free parameter, the slope, 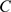.

We considered another model where the gain of postural reactions is initially constant, but decreases monotonically with increasing visual scene velocities (Fig. 3C). This model, with three free parameters, has the functional form:
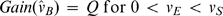


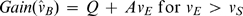



We fitted these models to the gain values of each subject tested in our experiment. We computed the Bayesian Information Criterion for each subject and for each model. We then performed a paired *t*-test to determine if there was a significant difference in the BIC values for different models.
